# Supplementary material for: Causal Relationship between Aging and Anorexia Nervosa: A White-Matter-Microstructure-Mediated Mendelian Randomization Analysis
Source: Biomedicines. 2024 Aug 16;12(8):1874. doi: 10.3390/biomedicines12081874 (PMC11351342; doi:10.3390/biomedicines12081874)
Supplement: Supplementary file 1 [file biomedicines-12-01874-s001.zip › Supplementary Figure/Figure S4/Singe SNP plot of MR analysis for TL to ubm-b-2036.pdf]

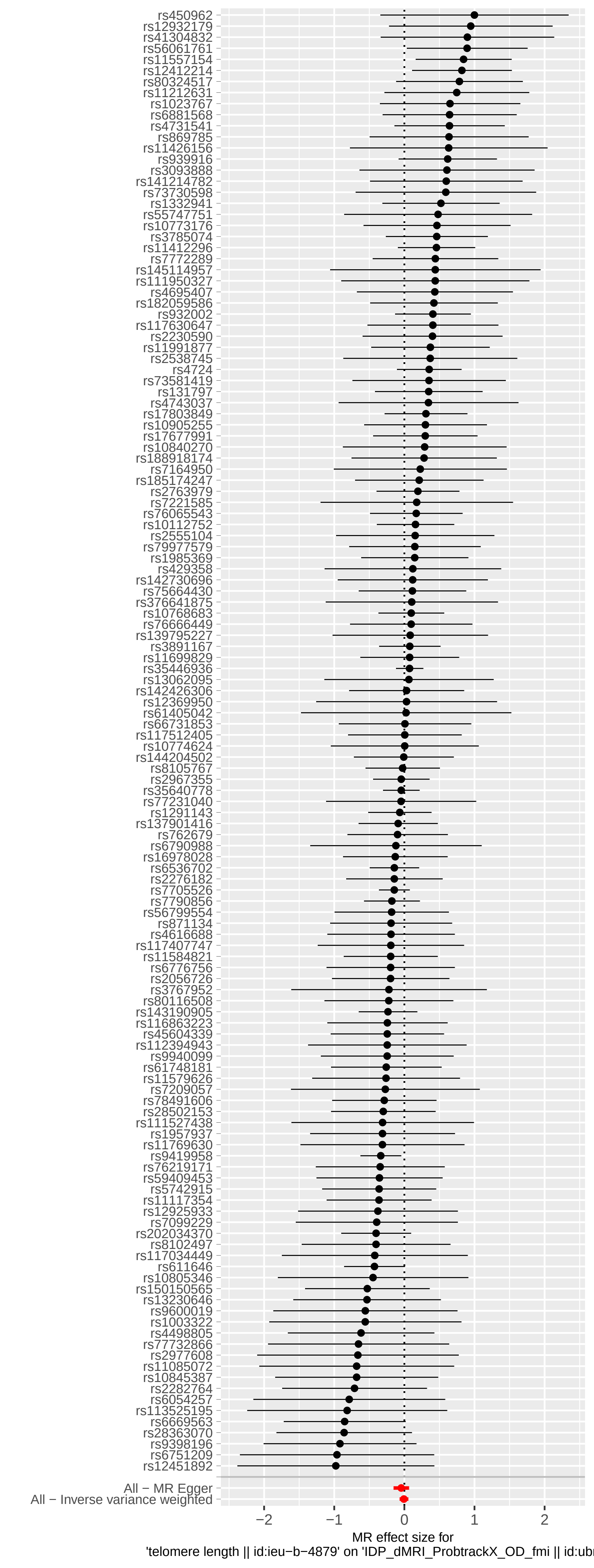

rs450962
rs12932179
rs41304832
rs56061761
rs11557154
rs12412214
rs80324517
rs11212631
rs1023767
rs6881568
rs4731541
rs869785
rs11426156
rs939916
rs3093888
rs141214782
rs73730598
rs1332941
rs55747751
rs10773176
rs3785074
rs11412296
rs7772289
rs145114957
rs111950327
rs4695407
rs182059586
rs932002
rs117630647
rs2230590
rs11991877
rs2538745
rs4724
rs73581419
rs131797
rs4743037
rs17803849
rs10905255
rs17677991
rs10840270
rs188918174
rs7164950
rs185174247
rs2763979
rs7221585
rs76065543
rs10112752
rs2555104
rs79977579
rs1985369
rs429358
rs142730696
rs75664430
rs376641875
rs10768683
rs76666449
rs139795227
rs3891167
rs11699829
rs35446936
rs13062095
rs142426306
rs12369950
rs61405042
rs66731853
rs117512405
rs10774624
rs144204502
rs8105767
rs2967355
rs35640778
rs77231040
rs1291143
rs137901416
rs762679
rs6790988
rs16978028
rs6536702
rs2276182
rs7705526
rs7790856
rs56799554
rs871134
rs4616688
rs117407747
rs11584821
rs6776756
rs2056726
rs3767952
rs80116508
rs143190905
rs116863223
rs45604339
rs112394943
rs9940099
rs61748181
rs11579626
rs7209057
rs78491606
rs28502153
rs111527438
rs1957937
rs11769630
rs9419958
rs76219171
rs59409453
rs5742915
rs11117354
rs12925933
rs7099229
rs202034370
rs8102497
rs117034449
rs611646
rs10805346
rs150150565
rs13230646
rs9600019
rs1003322
rs4498805
rs77732866
rs2977608
rs11085072
rs10845387
rs2282764
rs6054257
rs113525195
rs6669563
rs28363070
rs9398196
rs6751209
rs12451892
All − MR Egger
All − Inverse variance weighted
−2
−1
0
1
2
MR effect size for
'telomere length || id:ieu−b−4879' on 'IDP_dMRI_ProbtrackX_OD_fmi || id:ubm
